# Predictability of success and open conjunctival revision rates in the subsequent eye after XEN45 Gel Stent implantation according to lens status

**DOI:** 10.1007/s00417-022-05569-x

**Published:** 2022-02-03

**Authors:** D. Kiessling, C. Rennings, M. Hild, A. Lappas, T.S. Dietlein, G.F. Roessler, R.A. Widder

**Affiliations:** 1 Department of Ophthalmology, St. Martinus-Krankenhaus Düsseldorf, Gladbacher Str. 26, 40219 Düsseldorf, Germany; 2 Department of Ophthalmology, University Hospital of Cologne, Cologne, Germany; 3 Department of Ophthalmology, RWTH Aachen, Aachen, Germany

**Keywords:** Glaucoma incisional surgery, Microinvasive glaucoma surgery (MIGS), XEN, Microstent, Open-angle glaucoma

## Abstract

**Purpose:**

To determine the predictability of success and the risk of open conjunctival revision in the subsequent eye after XEN45 Gel Stent implantation according to lens status.

**Methods:**

This was a retrospective single-centre study involving 132 eyes of 66 participants who had undergone intraocular pressure (IOP)-lowering XEN45 Gel Stent implantation, either as a standalone procedure in phakic and pseudophakic eyes or in combination with phacoemulsification. Successful surgery was defined by three scores: IOP at follow-up < 21 mmHg (score A) or < 18 mmHg (score B) and an IOP reduction > 20% or IOP ≤ 15 mmHg and an IOP reduction ≥ 40% (score C). In all scores, one open conjunctival revision was allowed, and additional repeat surgery was considered a failure. The predictability of success and revision rate depending on the outcome of the first eye were calculated using Bayes’ theorem.

**Results:**

IOP-lowering did not differ significantly between the first and second eyes. Success rates of standalone surgery in the second eye after successful surgery in the first eye significantly exceed rates after prior failure. For the combined procedure, the rates did not differ significantly. For score A, we determined a 76.6% chance of success following a prior success and a 57.9% chance, if prior surgery failed. The corresponding probabilities were 75% and 59.1% for score B, while 66.7% and 15.7% for score C, respectively. We calculated a 60% risk for revision surgery in the standalone phakic group. If the first eye was not revised, the risk of revision in the subsequent eye was 20%. The corresponding risks were 72.7% and 5% for the standalone procedure in pseudophakic patients and 38.4% and 41.7% for the combined procedure, respectively.

**Conclusion:**

The results of our study offer a tool to predict the outcome of subsequent eye surgeries based on either the outcome in the initial eye and the type of surgery performed, owing to the high predictive potential.

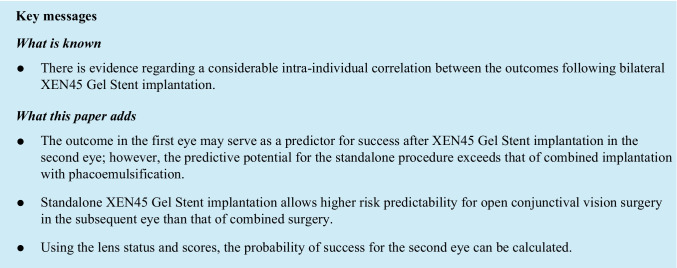



## Introduction

Being a primarily bilateral disease, glaucoma often requires surgical treatment on both eyes. Few studies have focused on whether the positive outcome of glaucoma surgery in the first eye has a predictive potential for success or clinical course influence in the subsequent eye. Mietz et al. reported that primary trabeculectomy in the fellow eye is associated with a greater risk of cyst formation in Tenon’s capsule [1].

In the past decades, traditional filtering surgeries, such as trabeculectomy or implantation of large lumen drainage devices, are the main treatment options for uncontrolled glaucoma. More recently, microinvasive glaucoma surgery (MIGS) has been used in routine clinical practice, in addition to established procedures. One of such is XEN45 Gel Stent implantation (Allergan, Dublin, CA, USA), which is a subconjunctival bleb-forming procedure that pursues an ab interno approach. Primary implantation is carried out via the anterior chamber through the trabecular meshwork and the scleral wall; hence, it does not require penetration of the conjunctiva. In addition to a moderate flow of aqueous humour through the small lumen, these factors contribute to a favourable safety profile, along with a promising intraocular pressure (IOP)-lowering potential [2–6].

In a recent study, Gillmann et al. determined considerable outcome predictability for the second eye based on the outcome after XEN45 Gel Stent implantation in the first eye, but they did not differentiate between the standalone and combined procedures [7].

However, secondary interventions are frequently needed because of postoperative scarring of the conjunctiva. Several studies have shown that combined stent implantation with phacoemulsification results in higher revision rates than the standalone procedure [2, 8]. This finding supports the question of whether phakic status or the combination with phacoemulsification exerts an influence on the predictive potential of successful XEN45 Gel Stent implantation in subsequent eyes, based on the outcome of the first operated eye.

For this reason, we investigated the outcomes between fellow eyes using different clinical scores to determine the predictive potential of the second eye surgery within groups of phakic or pseudophakic patients who underwent the standalone procedure and those who received combined stent implantation with phacoemulsification.

## Methods

This was a single-centre retrospective study based on the data acquired from the Department of Ophthalmology, St. Martinus-Krankenhaus, Düsseldorf, Germany. This study included all patients who underwent XEN45 Gel Stent implantation either as a standalone procedure or in combination with phacoemulsification, from 2015 to 2021. A total of 812 eyes of 737 patients were identified in our database, with 154 patients having received XEN45 Gel Stent implantation in both eyes. The inclusion criteria were an available baseline and a postoperative follow-up IOP value in patients undergoing XEN45 Gel Stent implantation in both eyes after at least 6 months and a maximum latency of 6 months between the surgery of the first and second eye. Eyes with secondary types of glaucoma, such as uveitic, neovascular, pigment dispersion, or dysgenetic glaucoma, were excluded (*n* = 6).

Considering the aforementioned criteria, 132 eyes of 66 patients remained in the study group, 122 were classified as having primary open-angle glaucoma (POAG), and 10 had exfoliation glaucoma (XFG). The epidemiological and baseline data are presented in Table 1.

**Table 1:** Epidemiology and baseline data

	All eyes (*n* = 132)	1st eyes (*n* = 66)	2nd eyes (*n* = 66)	*p*
Sex, *n* (%)				
Male	40 (30.3)	20 (30.3)	20 (30.3)	> *0.99*
Female	92 (69.7)	46 (69.7)	46 (69.7)	> *0.99*
Age (years)				
Mean ± SD	74.9 ± 9.9	74.9 ± 9.9	74.9 ± 9.9	> *0.99*
Baseline BCVA (logMAR)				
Mean ± SD	0.3 ± 0.3	0.3 ± 0.3	0.3 ± 0.3	*0.82*
Baseline C/D ratio				
Mean ± SD	0.8 ± 0.2	0.8 ± 0.2	0.8 ± 0.2	> *0.99*
Surgical procedure, *n* (%)				
Standalone phakic	20 (15.1)	10 (15.1)	10 (15.1)	> *0.99*
Standalone pseudophakic	62 (47.0)	31 (47.0)	31 (47.0)	> *0.99*
Combined w/ cataract surgery	50 (37.9)	25 (37.9)	25 (37.9)	> *0.99*
Glaucoma subtype, *n* (%)				
Primary open angle	122 (92.4)	61 (92.4)	61 (92.4)	> *0.99*
Exfoliation	10 (7.6)	5 (7.6)	5 (7.6)	> *0.99*
Baseline IOP (mmHg)				
All eyes	21.3 ± 4.8	21.9 ± 5.4	20.6 ± 3.7	*0.12*
Standalone phakic	20.6 ± 3.6	20.5 ± 3.5	20.6 ± 4.0	*0.95*
Standalone pseudophakic	22.4 ± 5.3	23.2 ± 6.4	21.6 ± 3.7	*0.24*
Combined w/ cataract surgery	20.1 ± 3.8	20.8 ± 4.2	19.4 ± 3.3	*0.21*
Follow-up IOP (mmHg)				
All eyes	14.6 ± 3.8	14.7 ± 4.1	14.4 ± 3.5	*0.59*
Standalone phakic	13.8 ± 2.8	13.8 ± 3.1	13.8 ± 2.7	> *0.99*
Standalone pseudophakic	14.3 ± 4.2	14.4 ± 4.1	14.2 ± 4.3	*0.86*
Combined w/ cataract surgery	15.1 ± 3.7	15.5 ± 4.5	14.8 ± 2.7	*0.50*
Highest preoperative IOP (mmHg)				
Mean ± SD	28.0 ± 6.9	28.3 ± 7.0	27.1 ± 4.9	*0.24*
Medication score at baseline				
Mean ± SD	2.3 ± 1.2	2.3 ± 1.2	2.3 ± 1.2	> *0.99*

*BCVA*, best-corrected visual acuity; *C/D*, cup/disc; *IOP*, intraocular pressure

*SD*, standard deviation

The *p* value was calculated via Student’s *t*-test for comparative analysis between 1st and 2nd eyes

The spectrum of prior eye surgery comprised selective laser trabeculoplasty (*n* = 27), ab interno trabeculectomy (*n* = 8), and 23 g pars plana vitrectomy (*n* = 2).

IOP was assessed using Goldmann applanation tonometry. A maximum of three IOP measurements prior to the date of the surgery were collected and averaged to evaluate the baseline IOP. Additional parameters included from the patients’ files were the type of glaucoma, maximum preoperative IOP, number of preoperative glaucoma medications, cup/disc ratio, and best-corrected visual acuity (BCVA) at baseline. The medication score comprised the amount of IOP-lowering medication classes applied at baseline and at follow-up.

The clinical endpoint at the longest follow-up examination was defined as either success or failure based on three separate scores. According to scores A and B, an absolute IOP < 21 mmHg (score A) or < 18 mmHg (score B) at follow-up examination and a postoperative IOP reduction of > 20% qualified for success. The two scores were chosen according to the tube versus trabeculectomy study [9]. The criteria for score C were an absolute IOP of 15 mmHg or less, and a postoperative IOP reduction of 40% or more, according to the criteria of the World Glaucoma Association [10]. In all scores, one open conjunctival revision was allowed. The aim of surgery was to lower the IOP without the use of medication; therefore, surgical revision was prioritised over repeat prescription of anti-glaucoma medication, unless the patient did not prefer additional surgery. Performing a second open conjunctival revision or repeat surgery was considered a failure.

### Surgical technique

Implantation of the XEN45 Gel Stent was performed as previously described [2, 11]. First, 0.1 ml of mitomycin C (0.1 mg/ml) was injected under the conjunctiva of the upper nasal quadrant, at a distance of 6 mm from the limbus. After temporal paracentesis, paracentesis at the 5 or 7 o’clock position, and anterior chamber stabilisation using a viscoelastic substance, the stent was placed using its injector device. The apex of the injector was driven through the trabecular meshwork and sclera at a distance of 3 mm from the limbus. The stent was then injected under the conjunctiva and the injector was removed. The position of the stent was confirmed via gonioscopy, and the viscoelastic substance was removed from the anterior chamber by irrigation.

For performing combined XEN45 Gel Stent implantation with phacoemulsification, a standard 2.5–2.8-mm clear corneal incision was made in the temporal cornea after the subconjunctival injection of mitomycin C. Two paracenteses were positioned at a 90° angle to the tunnel following bimanual phacoemulsification with posterior chamber and in-the-bag intraocular lens (IOL) implantation. Thereafter, stent implantation was performed as previously described [2, 11].

The aim of the surgery was to regulate the IOP without antiglaucomatous medication. Therefore, all patients without a sufficiently reduced IOP postoperatively underwent surgical revision. Instead of needling, we performed an open conjunctival approach, as described in our previous study [2, 12]. Briefly, the conjunctiva was incised at the limbus, the stent was located, and surrounding tissue was removed using microscissors and microforceps. After removal of scar tissues, the conjunctiva was refixated at the limbus with two absorbable 9.0 vicryl sutures (Coated Vicryl, Ethicon, New Brunswick, NJ, USA).

Standardised postoperative topical therapy was applied, including antibiotics (Floxal AS, Bausch & Lomb, Frankfurt, Germany) and steroid ointments (Ultracortenol AG, Agepha, Senec, Slovakia), both administered three times a day for 4 weeks. Previous topical anti-glaucomatous medications were discontinued.

### Ethics and statistics

The study and data collection were conducted with the approval of the institutional review board (Ethik und Kommission Klinische Studien, Dernbacher Gruppe Katharina Kasper, Germany). The tenets of the Declaration of Helsinki were regarded. Statistical analyses were performed using SPSS (version 24.0; IBM Corp. Armonk, NY, USA) and the statistical programming language R V3.2.2 (R Foundation for Statistical Computing, Vienna, Austria).

Outcomes in the first and second eyes, as well as the surgical success in subsequent eyes following either success or failure in the first eye, were compared using the log-rank test and visualised using Kaplan–Meier curves. Comparative analyses between mean IOP measurements at different time intervals as well as between preoperative and postoperative medication scores at follow-up were performed using Student’s *t*-test for paired samples. The chances of success in the subsequent eye after successful or failed surgery in the first eye as well as the risk for open conjunctival revision in the second eye after revision or absence of revision surgery in the first eye were calculated according to Bayes’ theorem. The use of the model for determining positive predictive values and conditional probabilities in clinical situations has been described in detail previously [13–15]. Statistical significance was set at *p* < 0.05.

## Results

### Baseline characteristics

The mean follow-up time interval was 17.6 ± 9.6 months in the first eye group and 15.6 ± 8.6 months in the second eye group (*p* = 0.22). During this follow-up period, XEN45 Gel Stent implantation significantly reduced the IOP (Table 1).

A total of 52 eyes (39%) underwent one open conjunctival revision, of which, three eyes (2%) required a second surgical revision using the same technique. Four of these eyes (3%) underwent additional glaucoma surgery at a later time point: trabeculectomy (*n* = 2) and cyclophotocoagulation (*n* = 2). In seven revised eyes (5%), intraoperative preparation of the stent showed a functional deficit, and the stent was replaced in the same surgical session.

### Survival analysis in the first and second eyes

**Fig. 1:**
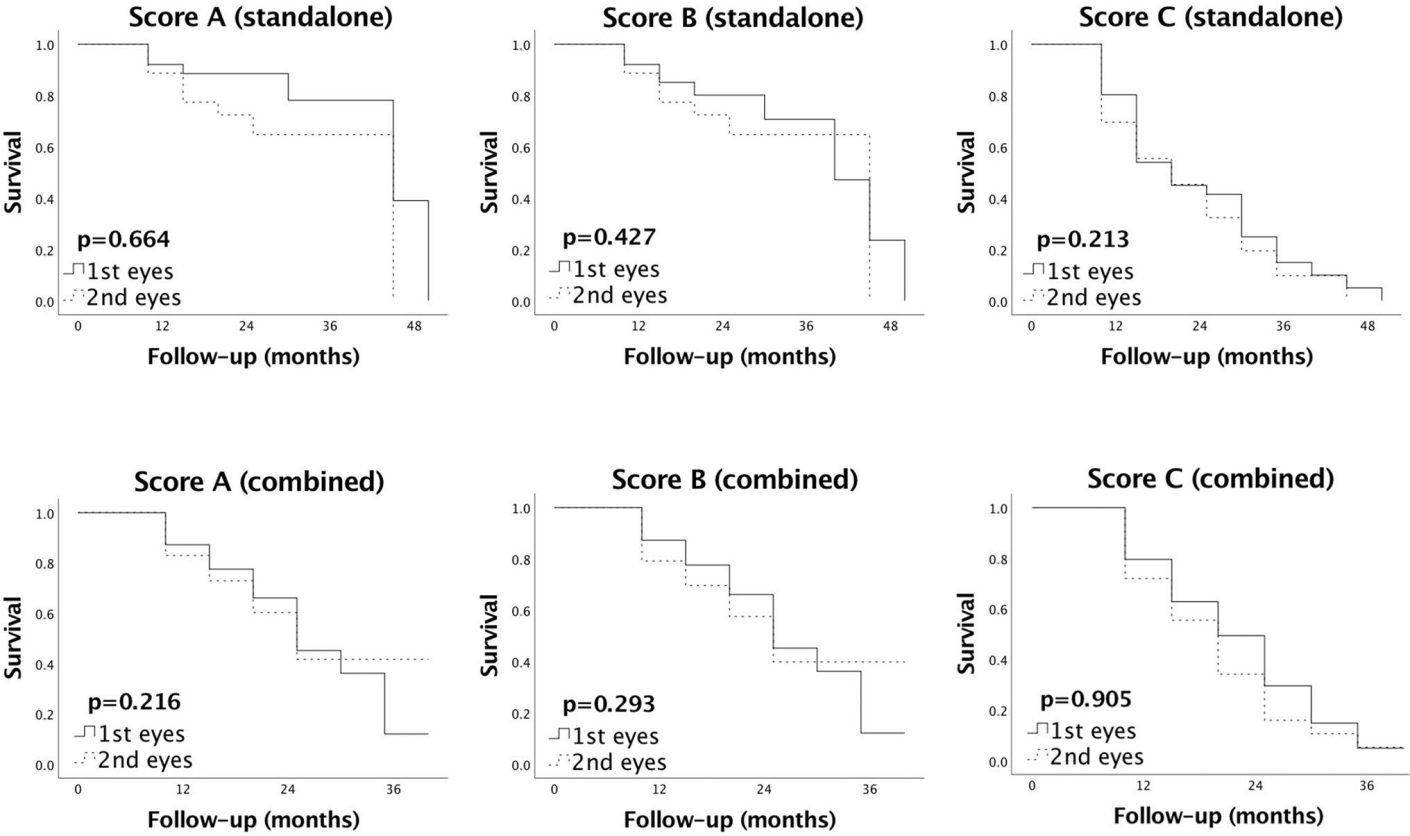
Kaplan–Meier survival curves comparing the success rates in the first and second eyes after standalone vs. combined XEN45 Gel Stent implantation. Score A: IOP at follow-up < 21 mmHg, IOP reduction > 20%, no repeat surgery. Score B: IOP at follow-up < 18 mmHg, IOP reduction > 20%, no repeat surgery. Score C: IOP at follow-up ≤ 15 mmHg, IOP reduction ≥ 40%, no repeat surgery

For both standalone and combined procedures, a comparative analysis between the outcome in the first and second eyes, with regard to the defined criteria for surgical success, did not show a significant difference. The Kaplan–Meier curves displayed corresponding trends, which remained parallel throughout the study (Fig. 1).

Mean preoperative IOP was 21.9 ± 5.4 mmHg in the first eye, which was higher than that of the second eye at 20.6 ± 3.7 mmHg and no significant difference was determined (*p* = 0.12).

In both groups, postoperative IOP values and medication scores remained stable at significantly lower levels throughout the follow-up timeframes at 6, 9, 12, 18, 24, 36, and 48 months, as the first and subsequent eyes did not show significant differences during follow-up (Table 2).

**Table 2:** Medication score and mean IOP (mmHg) at baseline and during follow-up

Time interval	*n*	Med score 1st eye	Med score 2nd eye	*p*	IOP 1st eye (mmHg)	IOP 2nd eye (mmHg)	*p*
Baseline	66	2.3 ± 1.2	2.3 ± 1.2	> *0.99*	21.9 ± 5.4	20.6 ± 3.7	*0.12*
6 months	19	0.1 ± 0.3	0.2 ± 0.4	*0.60*	13.6 ± 2.7	13.8 ± 2.8	*0.82*
9 months	23	0.1 ± 0.3	0.1 ± 0.3	> *0.99*	14.0 ± 1.9	14.2 ± 2.3	*0.84*
12 months	25	0.4 ± 0.7	0.2 ± 0.5	*0.22*	15.6 ± 7.2	15.5 ± 3.7	*0.94*
18 months	15	0.1 ± 0.5	0.0 ± 0.0	*0.34*	14.6 ± 3.5	14.0 ± 2.4	*0.59*
24 months	10	0.1 ± 0.3	0.1 ± 0.3	> *0.99*	13.7 ± 4.1	15.5 ± 6.6	*0.47*
36 months	10	0.5 ± 1.1	0.5 ± 1.1	> *0.99*	12.6 ± 2.8	14.4 ± 2.5	*0.15*
48 months	3	0.0 ± 0.0	1.0 ± 1.7	*0.37*	14.7 ± 4.9	15.3 ± 1.5	*0.83*

*IOP*, intraocular pressure; *p* value was calculated via Student’s *t*-test for comparative analysis between 1st and 2nd eyes

### Survival analysis in the subsequent eyes regarding the outcome in the first eyes

**Fig. 2:**
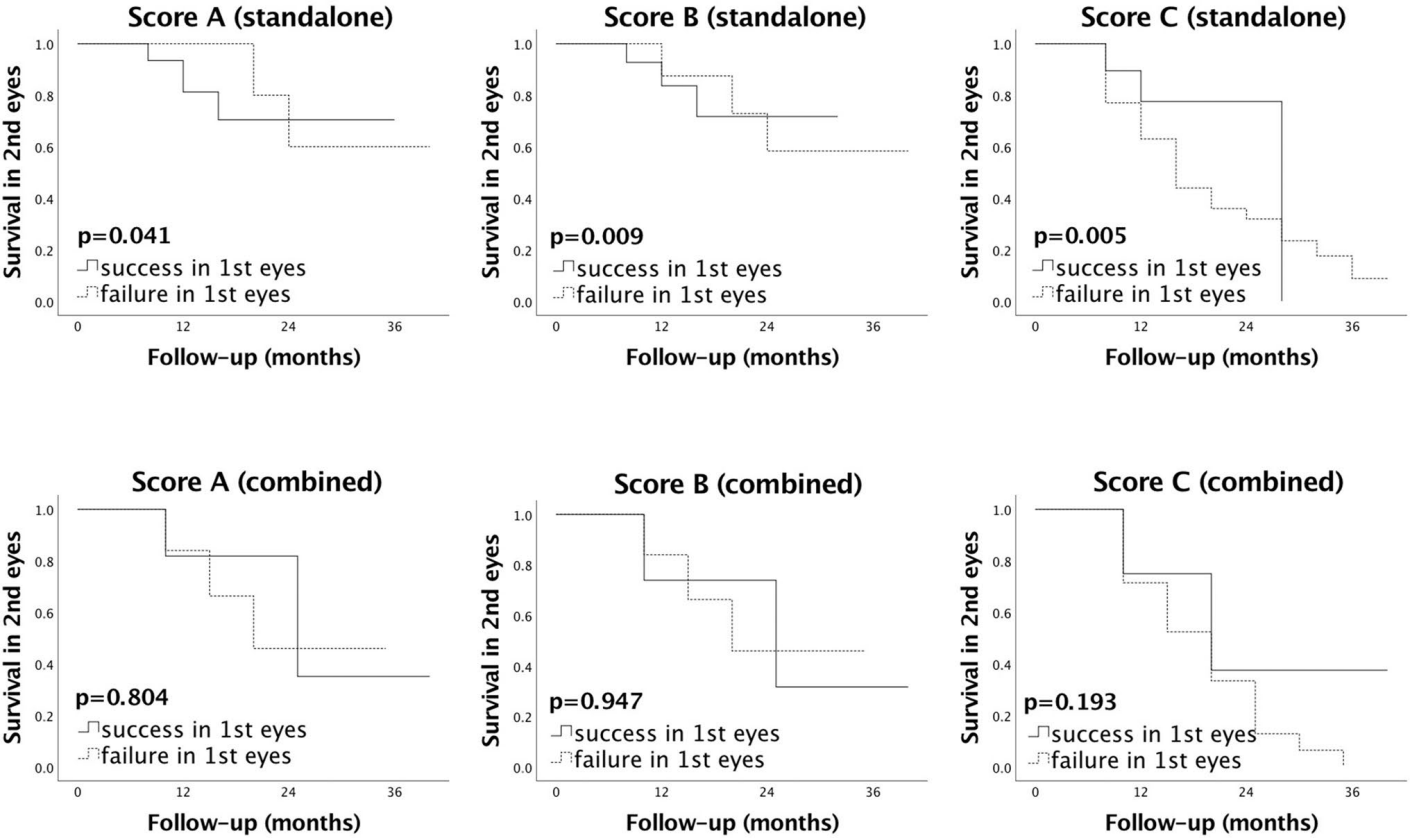
Kaplan–Meier survival curves comparing the success rates in the second eye after either successful or failed surgery of the first eye following standalone vs. combined XEN45 Gel Stent implantation. Score A: IOP at follow-up < 21 mmHg, IOP reduction > 20%, no repeat surgery. Score B: IOP at follow-up < 18 mmHg, IOP reduction > 20%, no repeat surgery. Score C: IOP at follow-up ≤ 15 mmHg, IOP reduction ≥ 40%, no repeat surgery

For standalone stent implantation survival analysis in the second eye, comparing cases with prior success or failure in the first eye determined a significant difference in the success rates for all scores (*p* < 0.05). Standalone stent implantation in the second eye after successful surgery in the first eye led to significantly better success rates than that in cases of prior failure (Fig. 2). In the combined group, there was no significant difference according to this criterion (*p* > 0.05).

### Probability calculations

According to Bayes’ theorem, which was used for probability calculation in determining the success and risk for open conjunctival revision in the second eye based on the outcome of the first eye, we found a 76.6% chance of success following a prior success for score A. However, if the first eye failed after surgery, the chance of success in the subsequent eye was reduced to 57.9%. The corresponding probabilities were 75% and 59.1% for score B and 66.7% and 15.7% for score C, respectively (Table 3).

**Table 3:** Chances of success in second eyes after either successful or failed surgery of the first eye after XEN45 Gel Stent implantation

Patients	No success in both eyes (*n*)	Success only in the 1st eye (*n*)	Success only in the 2nd eye (*n*)	Success in both eyes (*n*)	*Probability calculation *via* Bayes’s theorem*	
					Chance of success in the 2nd eye after success in the 1st eye (%)	Chance of success in the 2nd eye after failure in the 1st eye (%)
Score A (IOP < 21 mmHg, IOP reduction > 20%, one revision surgery allowed, no repeat surgery)						
All (*n* = 66)	8	11	11	36	76.6	57.9
Phakic (*n* = 10)	1	1	2	6	85.7	66.7
ps.ph. (*n* = 31)	2	6	2	21	77.8	50.0
comb. (*n* = 25)	5	4	7	9	69.2	58.3
Score B (IOP < 18 mmHg, IOP reduction > 20%, one revision surgery allowed, no repeat surgery)						
All (*n* = 66)	9	11	13	33	75.0	59.1
Phakic (*n* = 10)	1	1	2	6	85.7	66.7
ps.ph. (*n* = 31)	3	5	4	19	79.2	57.1
comb. (*n* = 25)	5	5	7	8	61.5	58.3
Score C (IOP ≤ 15 mmHg, IOP reduction ≥ 40%, one revision surgery allowed, no repeat surgery)						
All (*n* = 66)	43	5	8	10	66.7	15.7
Phakic (*n* = 10)	7	1	1	1	50.0	12.5
ps.ph. (*n* = 31)	17	2	6	6	75.0	26.1
comb. (*n* = 25)	19	2	1	3	60.0	5.0

*IOP*, intraocular pressure

Probability calculations determined a 60% risk for revision surgery following a prior revision of the first eye for the standalone procedure in phakic patients. If the first eye was not revised, the risk of revision in the subsequent eye was 20%. The corresponding probabilities were 72.7% and 5% for the standalone procedure in pseudophakic patients and 38.4% and 41.7% for the combined procedure, respectively (Table 4).

**Table 4:** Probability of revision surgery in second eyes after either revision or absence of revision in the first eye after XEN45 Gel Stent implantation

Patients	No revision in both eyes (*n*)	Revision only in the 1st eye (*n*)	Revision only in the 2nd eye (*n*)	Revision in both eyes (*n*)	*Probability calculation via Bayes’s theorem*		
					Probability of revision in the 2nd eye after revision in the 1st eye (%)	Probability of revision in the 2nd eye after absence of revision in the 1st eye (%)	
All (*n* = 66)	30	13	7	16	55.2		18.9
Phakic (*n* = 10)	4	2	1	3	60.0		20.0
Pseudophakic (*n* = 31)	19	3	1	8	72.7		5.0
Combined surgery (*n* = 25)	7	8	5	5	38.4		41.7

In the combined surgery group, the latter turned out to be relatively high, compared to the moderate chance of success for the second eye after prior success. In contrast, the range between both chances of success is considerably larger in cases that have undergone a standalone procedure.

### Side effects

Side effects included hyphema (*n* = 5), stent exposure (*n* = 2), cystoid macular edema (*n* = 1), and self-limited choroidal effusion due to transient hypotony (*n* = 1). Stent exposure required surgical revision and was counted as a re-surgery. No severe side effects were observed.

## Discussion

Consistent with previous studies, our study provided further evidence that XEN45 Gel Stent implantation effectively reduces both IOP and medication scores [2–4, 8].

Including only patients who underwent surgery in both eyes, we addressed three questions: First, does the outcome of the first eye influence the outcome of the second eye. Second, do intra-individual correlations differ between pseudophakic and phakic eyes or combined surgery, and third, does revision surgery in the first eye have a predictive value for revision surgery in the second eye.

Only a few studies have described the outcomes of the fellow eye after glaucoma surgery. Predictive values for trabecular aspiration and trabectome surgery have been reported [15, 16]. Upon filtering surgery investigations, Mietz et al. showed similar trends in the success rate of fellow eyes after trabeculectomy [1].

Recently, Gillmann et al. found a considerable intra-individual correlation between the outcomes following bilateral XEN45 Gel Stent implantation in a mixed cohort [7]. This study did not investigate differences between subgroups according to lens status and did not compare standalone surgery versus combined surgery.

Our results suggest that the predictability of success or revision rates is highly dependent on either phakic status and the combination with phacoemulsification at the time XEN45 Gel Stent implantation was performed. Predictability was higher when aiming at lower postoperative IOP values.

Considering the predictive potential for standalone surgery in second eyes derived from our data, we can conclude that, if surgical success is defined according to score A (IOP at follow-up < 21 mmHg, IOP reduction > 20%) and score B (IOP at follow-up < 18 mmHg, IOP reduction > 20%), XEN45 Gel Stent implantation for the subsequent eye still is a viable option after a failed first case. However, if the surgeon aims for lower IOP values for the second eye (score C: IOP at follow-up ≤ 15 mmHg, IOP reduction ≥ 40%), it might be reasonable to consider other filtering approaches.

Our results on the intra-individual correlation raise the question of whether there are factors that influence individual surgical outcomes. One of which might be individual susceptibility to subconjunctival scarring. In an extensive literature review, Broadway et al. listed unilateral predisposing issues, such as previous ocular surgery or secondary glaucoma due to trauma, uveitis, or neovascularisation, as well as bilateral risk factors. The latter include black skin colour, young age, and previous glaucoma medication when applied simultaneously. There is evidence that large amounts of benzalkonium chloride can cause drug-induced conjunctival inflammation and fibroblast proliferation, which is of crucial importance in the postoperative setting [17, 18].

Another factor that could act as an inter-individual variable in the surgical success rate is the lymphatics of the subconjunctival tissue, assuming that the function of drainage pathways is parallel among fellow eyes [19].

A reason for either diverging outcomes after standalone and combined surgery, as well as for a tendency toward more asymmetric outcomes among fellow eyes after the latter, could be found in the inflammatory response after surgery. Fellow-eye investigations after phacoemulsification have determined diverse concentrations of inflammatory chemokines in the aqueous humour of the first and second operated eyes as a reason for increased pain after second-eye surgery [20].

These molecular factors may contribute to the asymmetric response of IOP as a result of postoperative inflammation and subconjunctival scarring.

Combining traditional filtering surgery with phacoemulsification has been the subject of numerous studies. In the context of a meta-analysis, Friedman et al. found evidence that shows how phacotrabeculectomy results in less sufficient IOP reduction than standalone trabeculectomy [21].

It was postulated that increased trauma associated with a combined surgery leads to higher levels of transforming growth factor-beta 2 in the aqueous humour and prolonged alteration of the blood-aqueous barrier [22, 23]. These mechanisms might apply to combined XEN45 Gel Stent implantation with cataract surgery and the greater extent of its inter-individually varying outcomes compared to standalone surgery.

Notably, baseline IOP was lower in the combined surgery group than in both groups that received standalone surgery, which might have influenced a weaker relative IOP reduction. However, the mean postoperative IOP at the longest follow-up examination was markedly higher in the combined surgery group than in the standalone phakic and standalone pseudophakic group.

Moreover, the mean preoperative IOP in the second eye was significantly lower than that in the first eye. This might be brought about by the standard operating procedures of our department, which specifies that when the indication for XEN45 Gel Stent implantation is given for both eyes, surgery on the eye with the higher IOP is performed first, which in turn might have an impact on lower postoperative IOP values and higher success rates. The limitations of our study include its retrospective design, limited availability of follow-up data, and limited sample sizes among the three subgroups.

In summary, the results of our study offer a tool for glaucoma surgeons to estimate the success rates and risk of open conjunctival revision of the subsequent eye. We conclude that either the outcome of the first eye following XEN45 Gel Stent implantation or the type of surgery performed has an impact on the predictive potential and may help in decision-making when a second eye is considered for surgery.

## Abbreviations and acronyms

BCVA: Best-corrected visual acuity
C/D: Cup/disc
IOL: Intraocular lens
IOP: Intraocular pressure
MIGS: Microinvasive glaucoma surgery
POAG: Primary open-angle glaucoma
SD: Standard deviation
XFG: Exfoliation glaucoma
