# Correction to: Predictability of success and open conjunctival revision rates in the subsequent eye after XEN45 Gel Stent implantation according to lens status

**DOI:** 10.1007/s00417-022-05614-9

**Published:** 2022-03-03

**Authors:** D. Kiessling, C. Rennings, M. Hild, A. Lappas, T. S. Dietlein, G. F. Roessler, R. A. Widder

**Affiliations:** 1 Department of Ophthalmology, St. Martinus‑Krankenhaus Düsseldorf, Gladbacher Str. 26, 40219 Düsseldorf, Germany; 2 Department of Ophthalmology, University Hospital of Cologne, Cologne, Germany; 3 Department of Ophthalmology, RWTH Aachen, Aachen, Germany

**Correction to: Graefe's Archive for Clinical and Experimental Ophthalmology**

**https://doi.org/10.1007/s00417-022-05569-x**

In the published version of this article, the author names presentation is incorrect.

The correct presentation is provided below.

D Kiessling

C Rennings

M Hild

A Lappas

TS Dietlein

GF Roessler

RA Widder

This is being corrected in this publication.

